# Supplementary material for: Overcoming Preferred Orientation in Cryo-EM With Ultrasonic Excitation During Vitrification
Source: bioRxiv. 2025 Sep 17:2025.09.14.676144. Preprint. [Version 1] doi: 10.1101/2025.09.14.676144 (PMC12458302; doi:10.1101/2025.09.14.676144)
Supplement: Supplement 1 [file media-1.pdf]

Supplementary Information

Overcoming Preferred Orientation in Cryo-EM With Ultrasonic Excitation During Vitrification

Harry M. Williams[†a)], Wyatt A. Curtis[†a)], Michal Haubner[†a)], Jakub Hruby[†b)], Marcel Drabbels[b)], and Ulrich J. Lorenz[* a)]

Affiliations:

[a] Ecole Polytechnique Fédérale de Lausanne (EPFL), Laboratory of Molecular Nanodynamics, CH-1015 Lausanne, Switzerland

[b] Ecole Polytechnique Fédérale de Lausanne (EPFL), Laboratory for Ultrafast X-ray Sciences, CH-1015 Lausanne, Switzerland

[†]These authors contributed equally

* To whom correspondence should be addressed. Email: ulrich.lorenz@epfl.ch

This PDF file includes:

1 | Cryo-EM sample preparation

2 | Data processing and analysis – 50S ribosomal subunit

3 | Data processing and analysis – C-reactive protein

4 | Data processing and analysis – hemagglutinin

5 | Cryo-EM data collection and processing statistics

6 | Angular distribution in the conventional and ultrasonically excited hemagglutinin samples

7 | References

1 | Cryo-EM sample preparation

Cryo-EM samples are vitrified with a custom jet vitrification device that we integrated into a commercial plunge freezing apparatus (Thermo Fisher Vitrobot Mark IV).[1] Grids are prepared in the climate chamber of the plunge freezing apparatus. After single-sided blotting, the sample is lowered into the jet vitrification unit, which is mounted underneath the climate chamber. The sample is then excited with ultrasonic waves and vitrified with a jet of liquid ethane, after which the grid is immediately lowered further into a liquid ethane filled container that is placed underneath the jet vitrification device. From there, it is then transferred to liquid nitrogen and subsequently to a high-resolution electron microscope for imaging.

A purified sample of the 50S ribosomal subunit (40 OD_{260}/mL, 20 mM HEPES buffer, pH 7.5, 100 mM NaCl, 2 mM $MgCl_2$) was provided by Dr. Bertrand Beckert of the Dubochet Center for Imaging in Lausanne. Purified hemagglutinin was purchased from MyBioSource (MBS434205) in lyophilized form and resuspended in 1x PBS, pH 7.5 to a final concentration of 0.75 mg/mL. C-reactive protein from human fluids was purchased from Sigma-Aldrich (C4603) and used as is (2.6 mg/mL, 20 mM Tris, 280 mM NaCl, 5 mM $CaCl_2$, pH 7.8–8.2, 0.1% NaN_3).

Samples for jet vitrification are prepared in the climate chamber of the plunge freezing apparatus by applying 3.5 µL of the protein solution onto holey carbon grids (R1.2/1.3, 200 mesh copper, Quantifoil) that have been rendered hydrophilic (air glow discharge for 90 s, EasiGlow, Ted Pella) and clipped prior to use. After single-sided blotting (20 °C, 95% relative humidity, blotting force 0, 2 s blotting time for 50S ribosomal subunit; 4 °C, 90% relative humidity, blotting force 10, 4.5 s blotting time for hemagglutinin; 20 °C, 95% relative humidity, blotting force 0, 3.5 s blotting time for the C-reactive protein), the sample is then lowered into the jet vitrification device.

Samples are vitrified with a jet of liquid ethane. The jet is created by condensing ethane gas into a brass reservoir cooled to 98 K and then expelling the cryogenic liquid (about 0.3 mL) through a tube (2 mm inner diameter) with a pulse of compressed nitrogen. The jet travels with a speed of about 2.6 m/s when it reaches the sample. The sample is then immediately lowered further into a liquid ethane filled container. To improve the angular distribution of the particles, the grids are mechanically excited with

ultrasonic waves for about 10 ms prior to the arrival of the liquid ethane jet. To this end, an ultrasonic transducer (SECO SC049) is placed at a distance of 9 mm from the sample and is excited with a 490 kHz square wave of 100 V amplitude, providing an estimated sound intensity level of about 126 dB at the sample location.

2 | Data processing and analysis – 50S ribosomal subunit

High-resolution micrographs of a conventional cryo sample of the 50S ribosomal subunit as well as a sample that was subjected to ultrasonic excitation were collected at the Dubochet Center for Imaging in Lausanne, using a Thermo Fisher Titan Krios G4i transmission electron microscope equipped with a Falcon IV direct electron detector. Single-particle reconstructions were performed using CryoSPARC v4.7.1[2], with the corresponding workflows illustrated in SI Figures 1 and 2. The data acquisition parameters and processing statistics are summarized in SI Table 1.

Micrographs were subjected to patch motion correction and patch CTF estimation. Micrographs with an estimated resolution of worse than 10 Å were rejected. Particles from the conventional and ultrasonically excited samples were picked using a blob picker with a diameter ranging from 150 to 400 Å. The particles were extracted with a box size of 784 px and Fourier-cropped to 196 px.

For the conventional dataset, particles were first subjected to three rounds of 2D classification followed by *ab initio* reconstruction (three classes, C_1 symmetry). Particles from the two best *ab initio* classes were homogeneously refined (C_1 symmetry) into a single volume. This volume and two decoys were then used in two rounds of heterogeneous refinement (three classes, C_1 symmetry). Particles from the best class resulting from the final round of heterogeneous refinement were re-extracted with a box size of 784 px. A random subset of 50,000 of these re-extracted particles were then homogeneously refined (C_1 symmetry) producing the final volume shown in SI Figure 1.

For the ultrasonically excited dataset, particles were subjected to three rounds of 2D classification followed by *ab initio* reconstruction (three classes, C_1 symmetry). Particles from the best *ab initio* classes were homogeneously refined (C_1 symmetry) into a single volume. This volume and two decoys were then used in two rounds of heterogeneous refinement (three classes, C_1 symmetry). Particles from the best class resulting from the final round of heterogeneous refinement were re-extracted with a box size of 784 px. A random subset of 50,000 of these re-extracted particles were homogeneously refined (C_1 symmetry) producing the final volume shown in SI Figure 2.

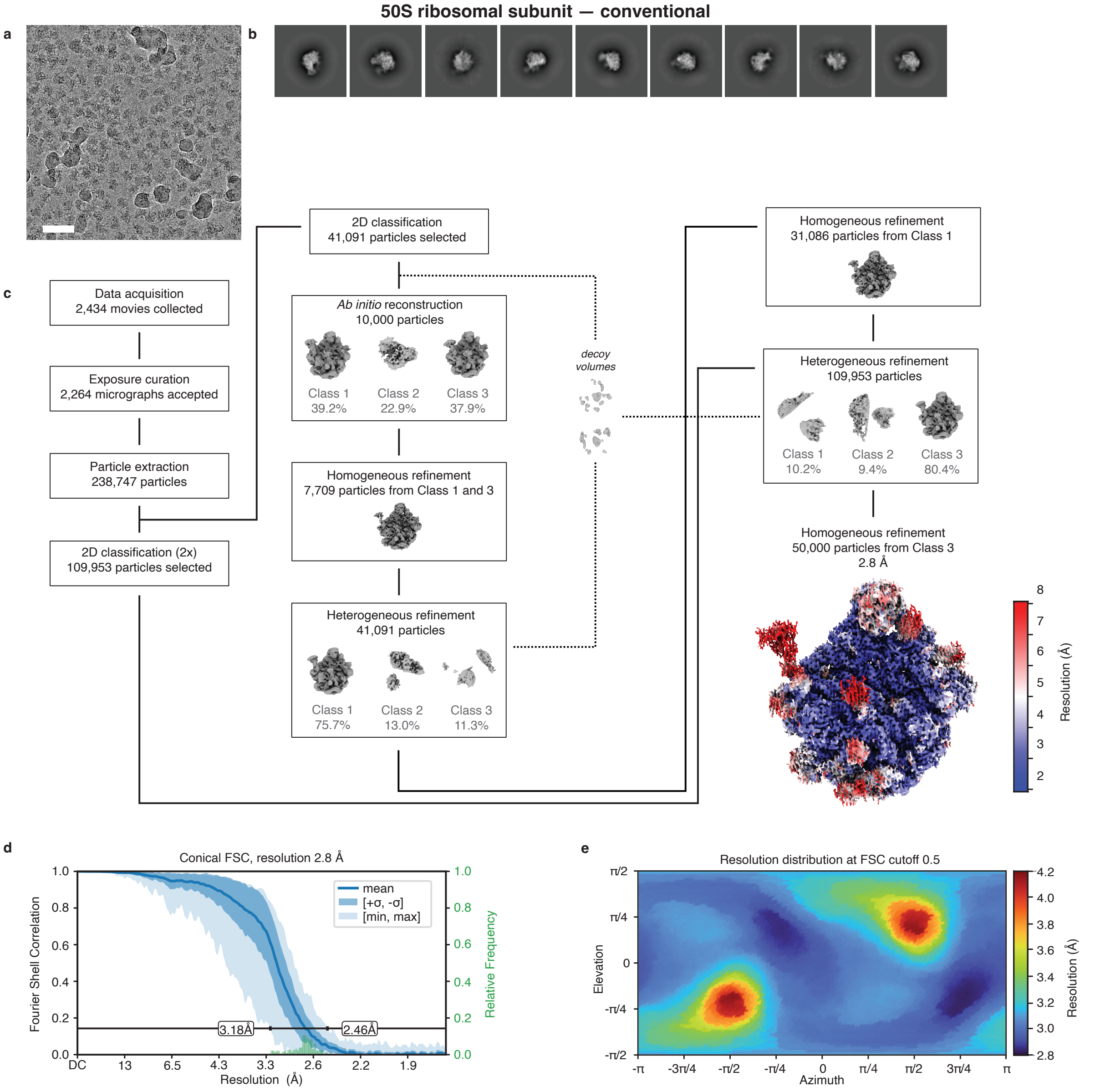

Figure S1 | Processing workflow for the conventional sample of the 50S ribosomal subunit. **a** Representative micrograph. Scale bar, 500 Å. **b** Selected 2D class averages. **c** Data processing workflow in CryoSPARC. All reconstructions are performed with C_1 symmetry. The volumes are displayed at a level of 5σ above the mean. The final map is displayed at a level 3σ above the mean, with the local resolution estimation at the FSC cutoff of 0.5 indicated in color. **d** Conical FSC, with the 0.143 threshold indicated by a black line. The mean FSC value is shown as a solid blue line. Dark blue shading indicates one standard deviation of the FSC curve, while the light blue shading represents the minimum and maximum FSC values. A histogram of the resolution values obtained from the conical FSC is shown in green. **e** Angular distribution of the resolution at FSC cutoff 0.5, generated using the 3DFSC file from Orientation Diagnostics job in CryoSPARC.

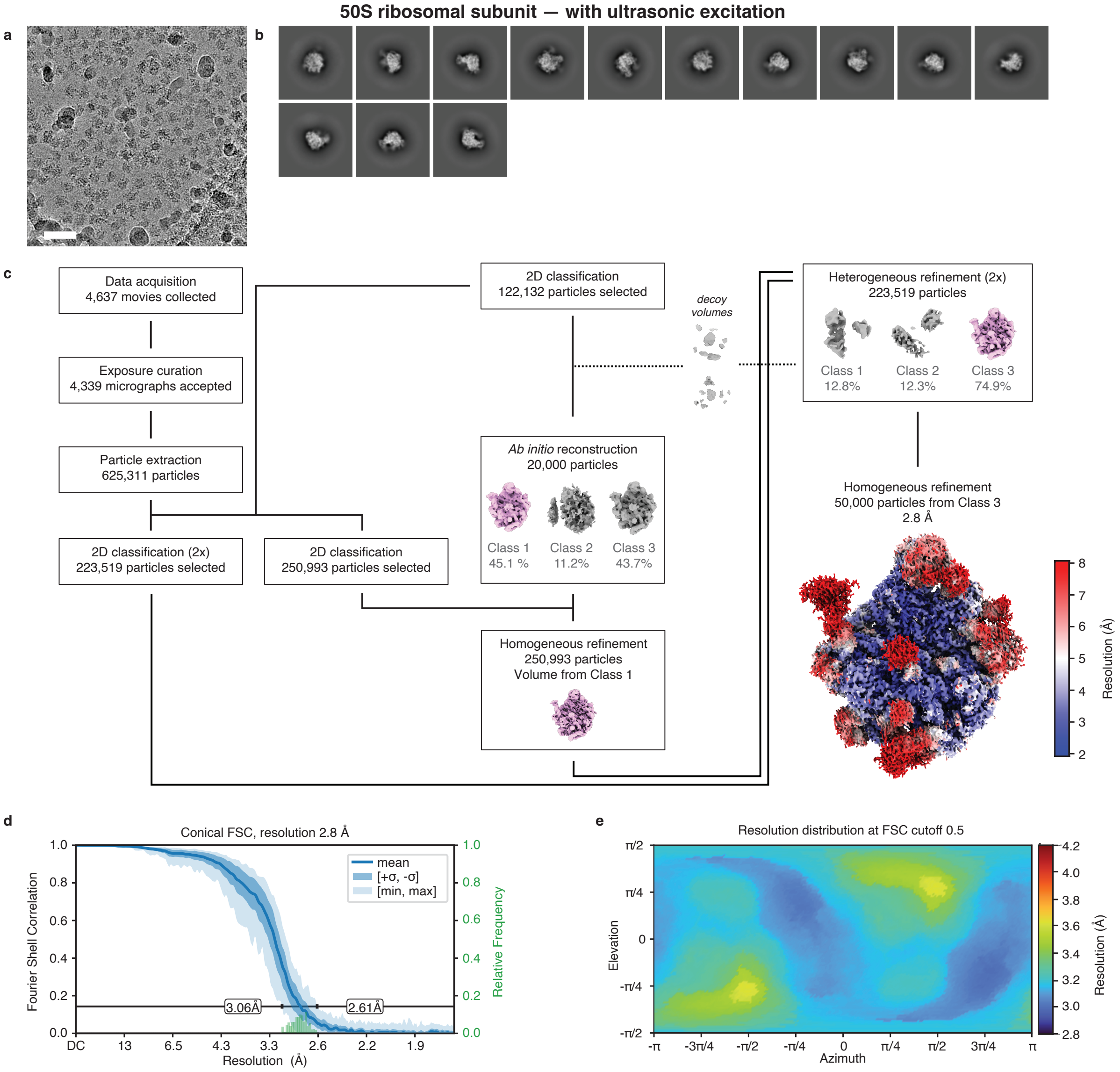

Figure S2 | Processing workflow for the ultrasonically excited sample of the 50S ribosomal subunit. **a** Representative micrograph. Scale bar, 500 Å. **b** Selected 2D class averages. **c** Data processing workflow in CryoSPARC. All reconstructions are performed with C_1 symmetry. The volumes are displayed at a level of 5σ above the mean. The final map is displayed at a level 3σ above the mean, with the local resolution estimation at the FSC cutoff of 0.5 indicated in color. **d** Conical FSC, with the 0.143 threshold indicated by a black line. The mean FSC value is shown as a solid blue line. Dark blue shading indicates one standard deviation of the FSC curve, while the light blue shading represents the minimum and maximum FSC values. A histogram of the resolution values obtained from the conical FSC is shown in green. **e** Angular distribution of the resolution at FSC cutoff 0.5, generated using the 3DFSC file from Orientation Diagnostics job in CryoSPARC.

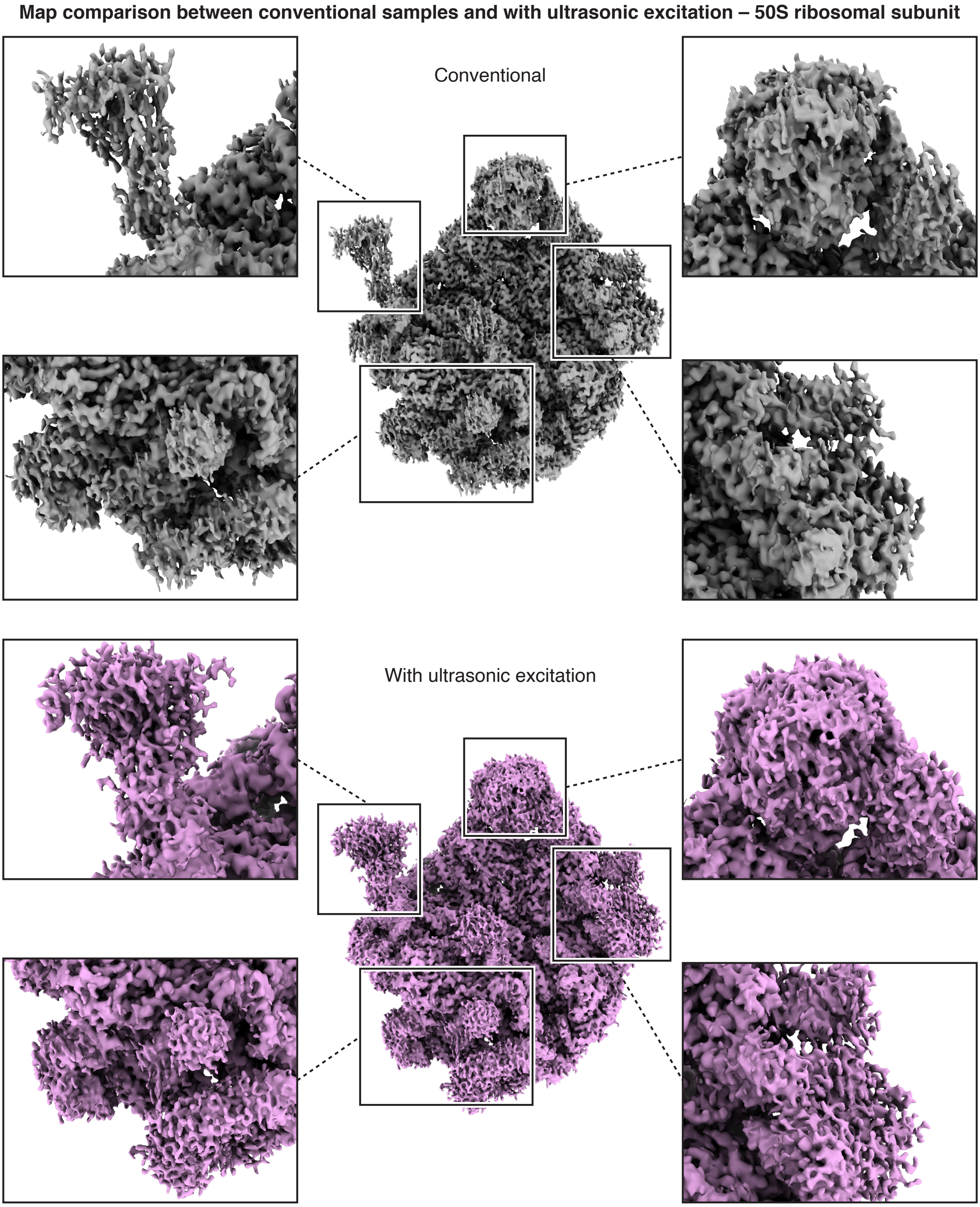

Figure S3 | Comparison of the reconstructions from the conventional and ultrasonically excited samples of the 50S ribosomal subunit. The streaky artefacts visible in the reconstruction from the conventional sample are caused by preferred orientation but are reduced with ultrasonic excitation. All maps are displayed at a level of 3σ above the mean.

3 | Data processing and analysis – C-reactive protein

High-resolution micrographs of a conventional cryo sample of human C-reactive protein as well as a sample that was subjected to ultrasonic excitation were collected at the Dubochet Center for Imaging in Lausanne, using a Thermo Fisher Titan Krios G4i transmission electron microscope equipped with a Falcon IV direct electron detector. Single-particle reconstructions were performed using CryoSPARC v4.7.1[2], with the corresponding workflows illustrated in SI Figures 4-7. The data acquisition parameters and processing statistics are summarized in SI Table 2.

Micrographs were subjected to patch motion correction and patch CTF estimation. Micrographs with an estimated resolution of worse than 10 Å were rejected. Particles from the conventional and ultrasonically excited samples were picked using a blob picker with a diameter ranging from 20 to 130 Å. The particles were extracted with a box size of 384 px and Fourier-cropped to 96 px.

For the conventional dataset, particles were first subjected to six rounds of 2D classification during which particles corresponding to the C-reactive protein pentamer and decamer are separated. After 2D classification, particles corresponding to the conventional pentamer were subjected to *ab initio* reconstruction (one class, C_1 symmetry), homogeneous refinement (C_5 symmetry), and heterogeneous refinement (four classes, C_5 symmetry) using the previously homogeneously refined volume and three decoy volumes. Particles from the best class were then subjected to two rounds of 2D classification and re-extracted with a box size of 384 px. *Ab initio* reconstruction was then used on these remaining particles to produce three volumes (C_5 symmetry), each of which was then homogeneously refined (C_5 symmetry). Particles from the best two classes were subjected to a final round of heterogeneous refinement (four classes, C_5 symmetry). A random subset of 30,000 particles corresponding to the best volume in the prior heterogeneous refinement job were non-uniformly refined (C_5 symmetry) producing the final volume shown in SI Figure 4.

Similarly, following 2D classification, particles corresponding to the conventional decamer were subjected to *ab initio* reconstruction (three classes, C_1 symmetry), homogeneous refinement (C_1 symmetry), and heterogeneous refinement (five classes, C_1 symmetry) using the previously homogeneously refined volume and four decoy volumes. Particles from the best class were then

subjected to two rounds of 2D classification and re-extracted with a box size of 384 px. *Ab initio* reconstruction was then used on these remaining particles to produce three volumes (C_1 symmetry), each of which was then homogeneously refined (C_1 symmetry). Particles from the best two classes were subjected to a final round of heterogeneous refinement (four classes, C_1 symmetry). A random subset of 50,000 particles corresponding to the best volume in the prior heterogeneous refinement job were non-uniformly refined (C_1 symmetry) producing the final volume shown in SI Figure 6.

The ultrasonically excited dataset was processed similarly. Particles were first subjected to six rounds of 2D classification after which particles corresponding to the ultrasonically excited pentamer were subjected to *ab initio* reconstruction (one class, C_1 symmetry), homogeneous refinement (C_5 symmetry), and heterogeneous refinement (five classes, C_5 symmetry) using the previously homogeneously refined volume and four decoy volumes. Particles from the best class were then subjected to two rounds of 2D classification and re-extracted with a box size of 384 px. *Ab initio* reconstruction was then used on these remaining particles to produce three volumes (C_5 symmetry), each of which was then homogeneously refined (C_5 symmetry). Particles from the best two classes were subjected to a final round of heterogeneous refinement (four classes, C_5 symmetry). A random subset of 30,000 particles corresponding to the best volume in the prior heterogeneous refinement job were non-uniformly refined (C_5 symmetry) producing the final volume shown in SI Figure 5.

Following 2D classification, particles corresponding to the ultrasonically excited decamer were subjected to *ab initio* reconstruction (three classes, C_1 symmetry), homogeneous refinement (C_1 symmetry), and heterogeneous refinement (four classes, C_1 symmetry) using the previously homogeneously refined volume and three decoy volumes. Particles from the best class were then subjected to two rounds of 2D classification and re-extracted with a box size of 384 px. *Ab initio* reconstruction was then used on these remaining particles to produce three volumes (C_1 symmetry), each of which was then homogeneously refined (C_1 symmetry). Particles from the best two classes were subjected to a final round of heterogeneous refinement (four classes, C_1 symmetry). A random subset of 50,000 particles corresponding to the best volume in the prior heterogeneous refinement job were non-uniformly refined (C_1 symmetry) producing the final volume shown in SI Figure 7.

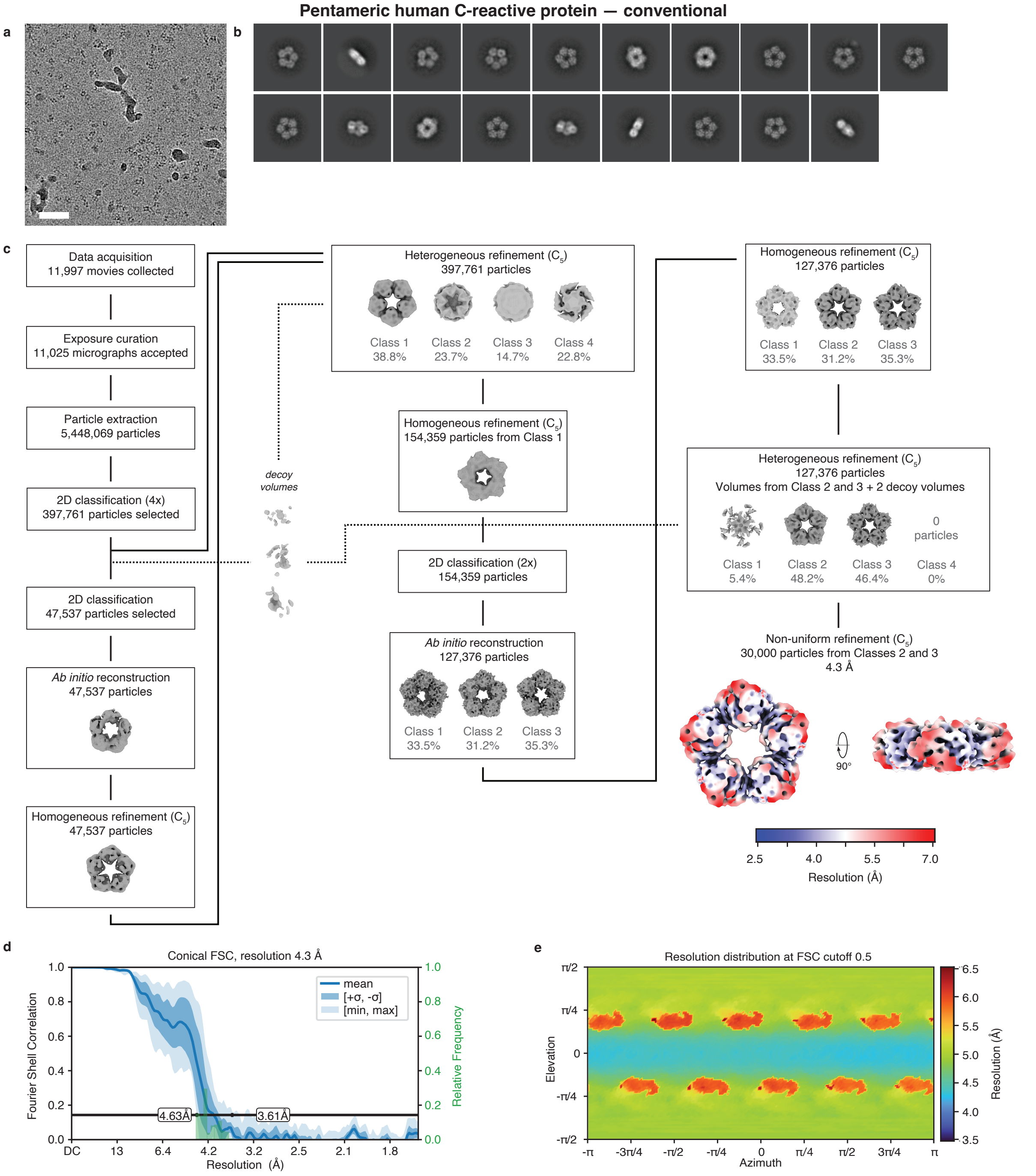

Figure S4 | Processing workflow for the conventional sample of the C-reactive protein pentamer. **a** Representative micrograph. Scale bar, 500 Å. **b** Selected 2D class averages. **c** Data processing workflow in CryoSPARC. The symmetry applied in each step is indicated in parentheses. The volumes are displayed at a level of 7σ above the mean. The final map is displayed at a level 10σ above the mean, with the local resolution estimation at the FSC cutoff of 0.5 indicated in color. **d** Conical FSC, with the 0.143 threshold indicated by a black line. The mean FSC value is shown as a solid blue line. Dark blue shading indicates one standard deviation of the FSC curve, while the light blue shading

represents the minimum and maximum FSC values. A histogram of the resolution values obtained from the conical FSC is shown in green. **e** Angular distribution of the resolution at FSC cutoff 0.5, generated using the 3DFSC file from Orientation Diagnostics job in CryoSPARC.

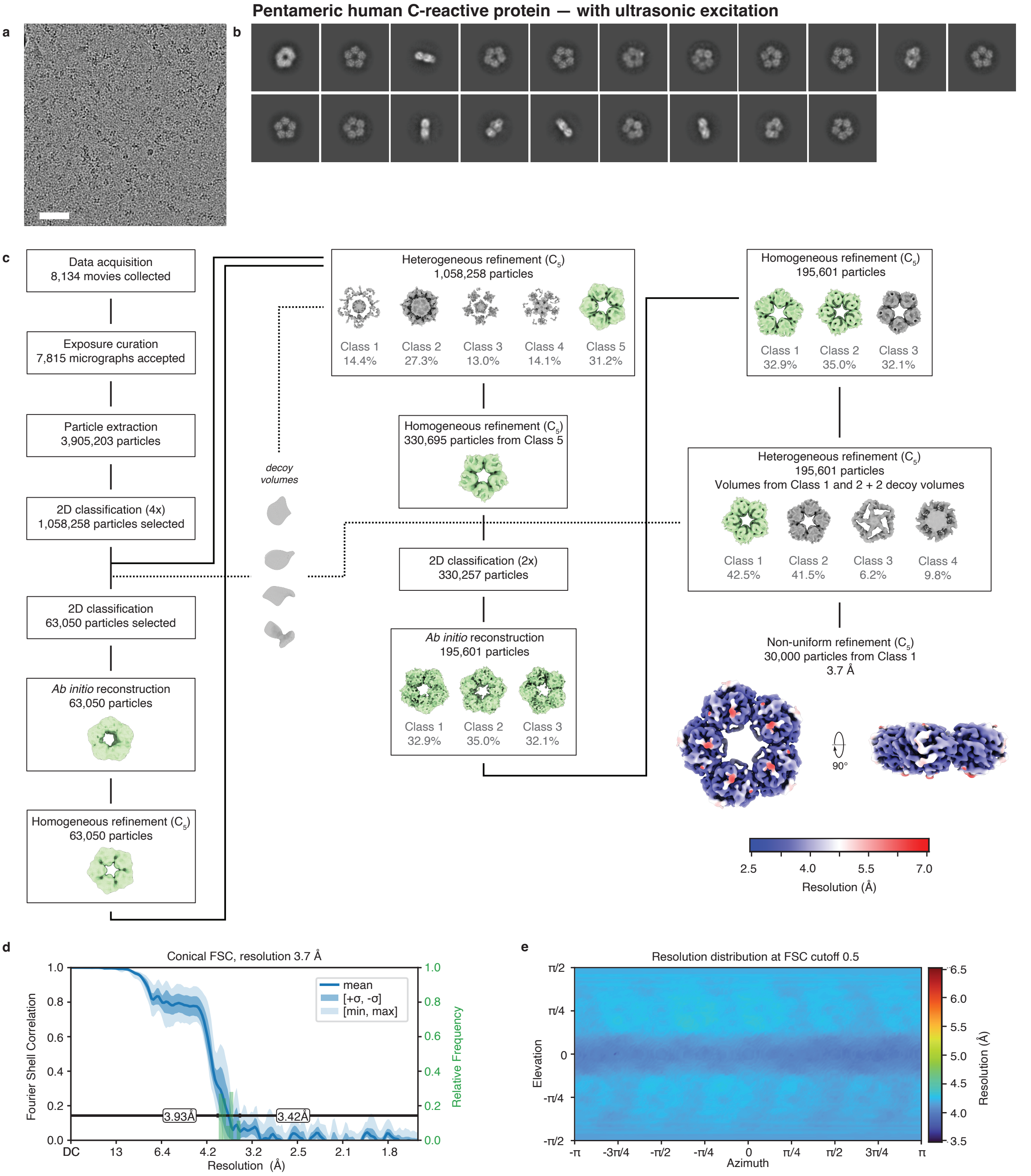

Figure S5 | Processing workflow for the ultrasonically excited sample of the C-reactive protein pentamer. a Representative micrograph. Scale bar, 500 Å. **b** Selected 2D class averages. **c** Data processing workflow in CryoSPARC. The symmetry applied in each step is indicated in parentheses. The volumes are displayed at a level of 7σ above the mean. The final map is displayed at a level 10σ above the mean, with the local resolution estimation at the FSC cutoff of 0.5 indicated in color. **d** Conical FSC, with the 0.143 threshold indicated by a black line. The mean FSC value is shown as a solid blue line. Dark blue shading indicates one standard deviation of the FSC curve, while the light blue shading

represents the minimum and maximum FSC values. A histogram of the resolution values obtained from the conical FSC is shown in green. **e** Angular distribution of the resolution at FSC cutoff 0.5, generated using the 3DFSC file from Orientation Diagnostics job in CryoSPARC.

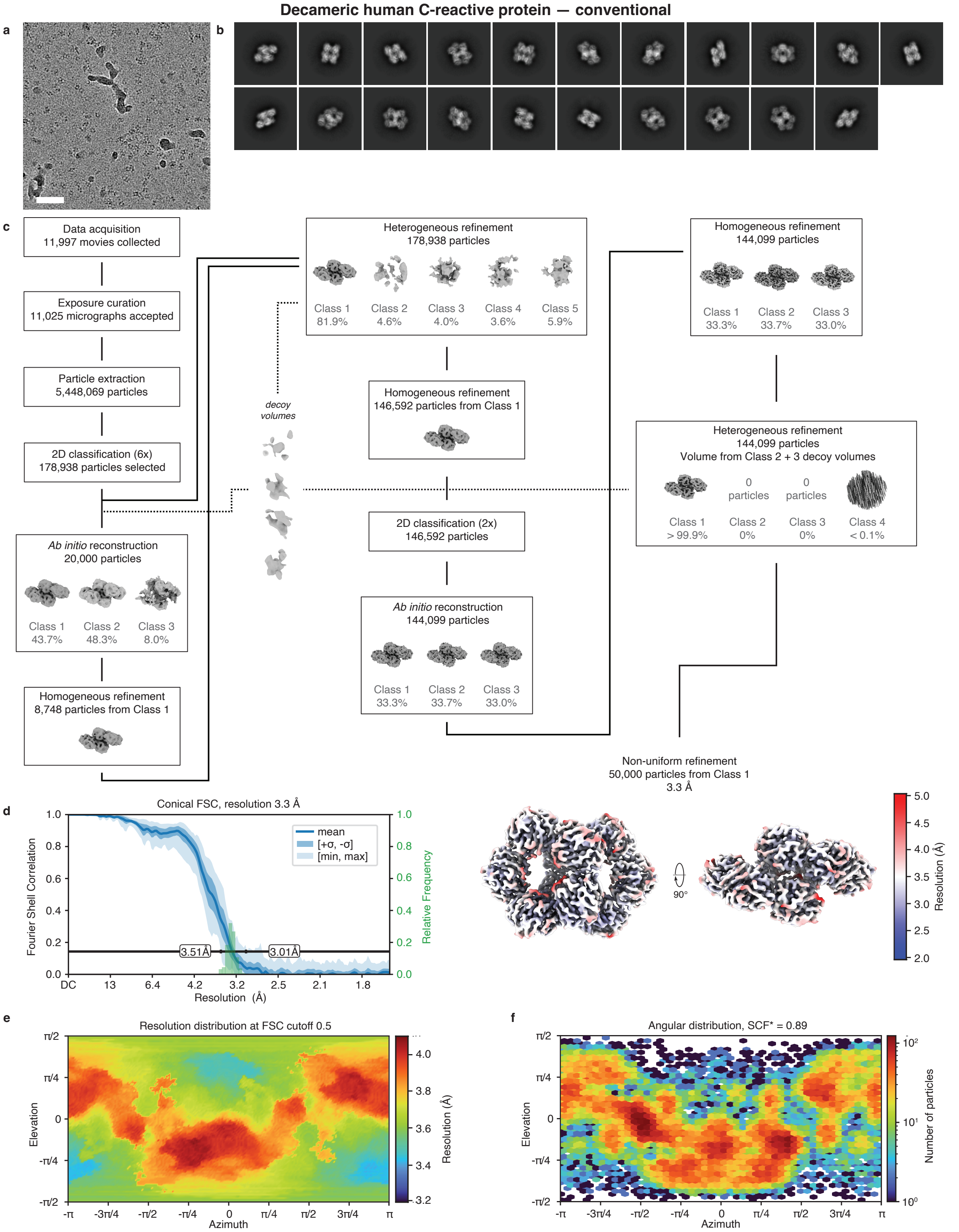

Figure S6 | Processing workflow for the conventional sample of the C-reactive protein decamer. **a** Representative micrograph. Scale bar, 500 Å. **b** Selected 2D class averages. **c** Data processing workflow in CryoSPARC. All reconstructions are performed with C_1 symmetry. The volumes are displayed at a level of 7σ above the mean. The final map is displayed at a level 10σ above the mean,

with the local resolution estimation at the FSC cutoff of 0.5 indicated in color. **d** Conical FSC, with the 0.143 threshold indicated by a black line. The mean FSC value is shown as a solid blue line. Dark blue shading indicates one standard deviation of the FSC curve, while the light blue shading represents the minimum and maximum FSC values. A histogram of the resolution values obtained from the conical FSC is shown in green. **e** Angular distribution of the resolution at FSC cutoff 0.5, generated using the 3DFSC file from Orientation Diagnostics job in CryoSPARC. **f** Orientation distribution of the particles in the indicated final volume.

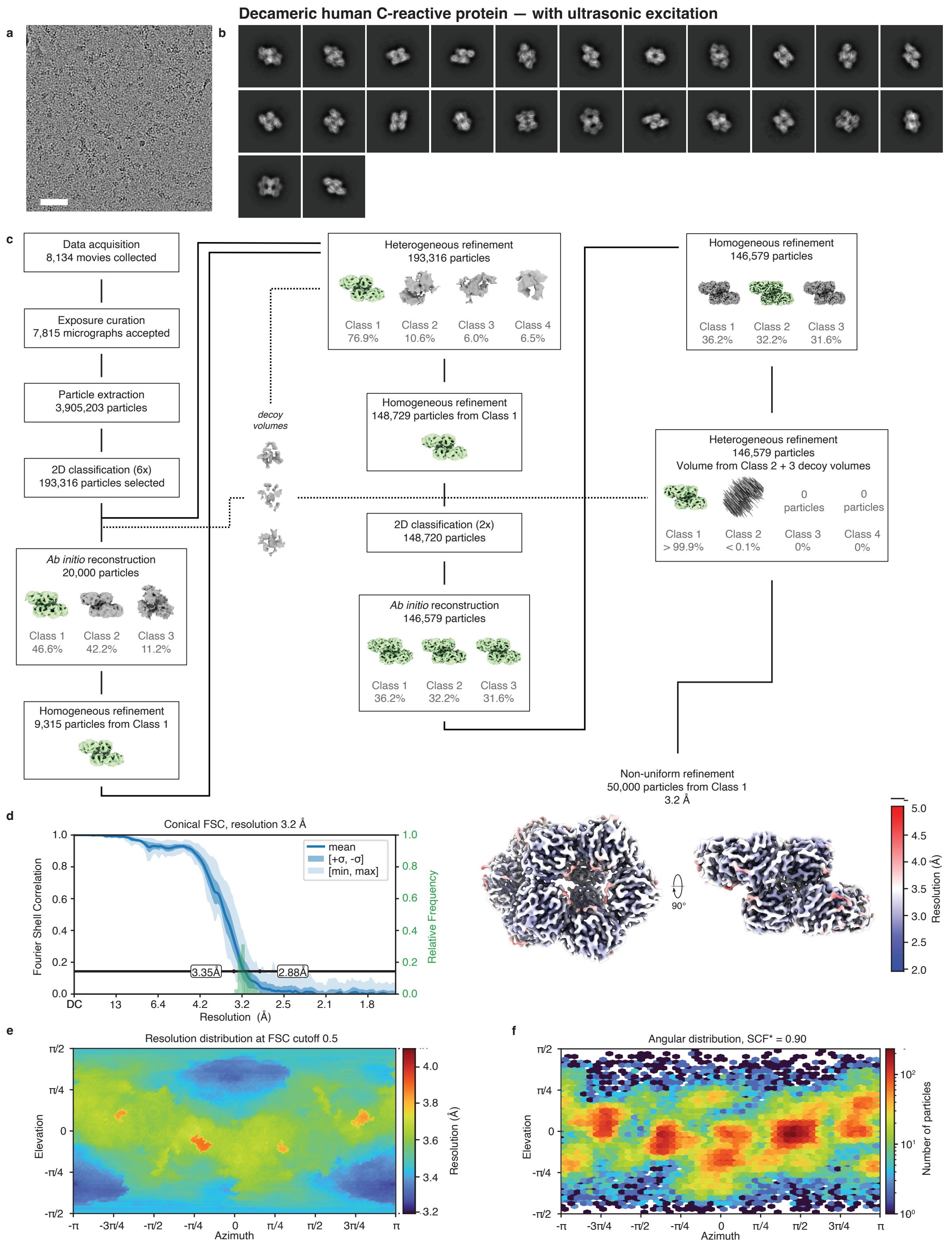

Figure S7 | Processing workflow for the ultrasonically excited sample of the C-reactive protein decamer. **a** Representative micrograph. Scale bar, 500 Å. **b** Selected 2D class averages. **c** Data processing workflow in CryoSPARC. All reconstructions are performed with C_1 symmetry. The volumes are displayed at a level of 7σ above the mean. The final map is displayed at a level 10σ above the

mean, with the local resolution estimation at the FSC cutoff of 0.5 indicated in color. **d** Conical FSC, with the 0.143 threshold indicated by a black line. The mean FSC value is shown as a solid blue line. Dark blue shading indicates one standard deviation of the FSC curve, while the light blue shading represents the minimum and maximum FSC values. A histogram of the resolution values obtained from the conical FSC is shown in green. **e** Angular distribution of the resolution at FSC cutoff 0.5, generated using the 3DFSC file from Orientation Diagnostics job in CryoSPARC. **f** Orientation distribution of the particles in the indicated final volume.

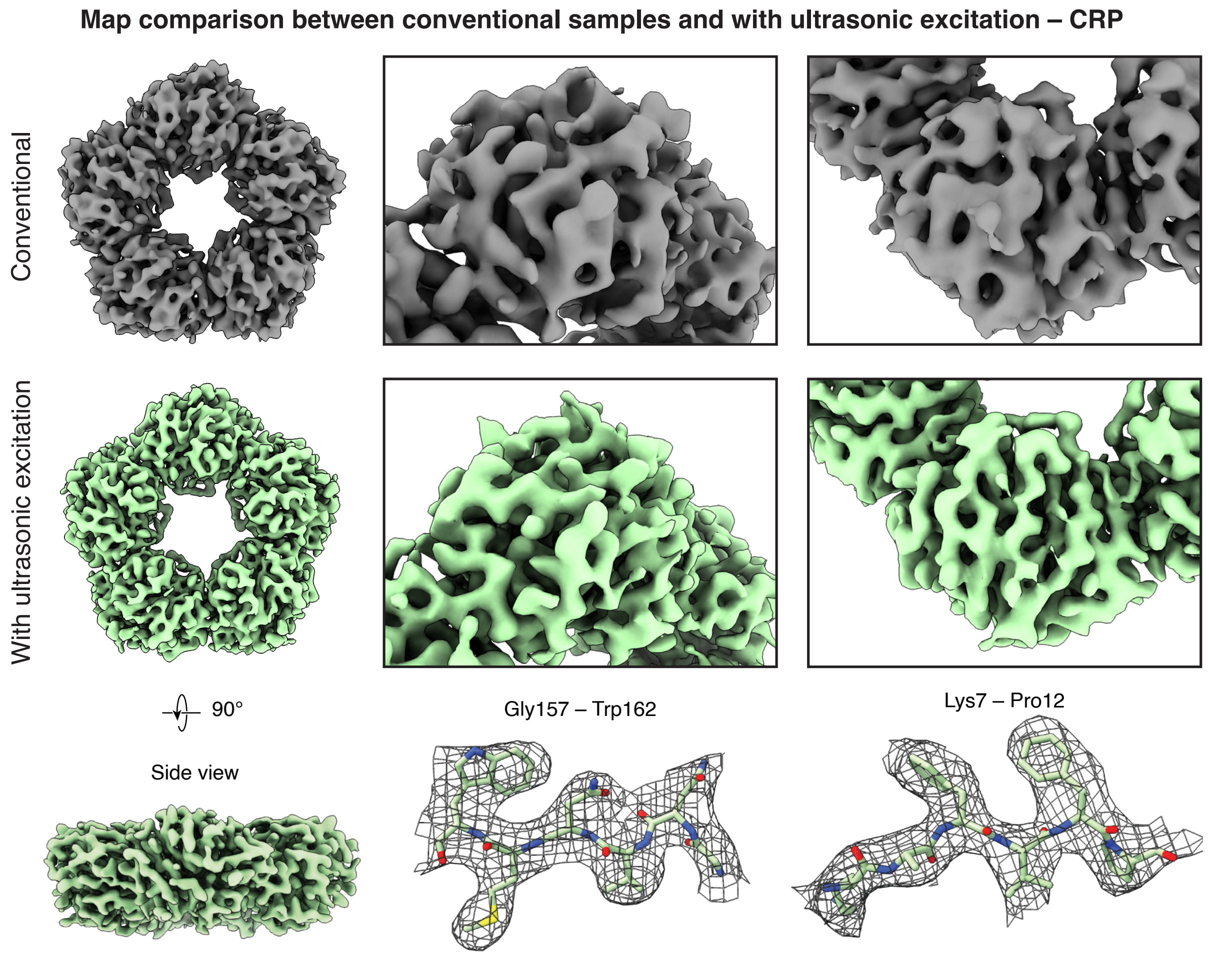

Figure S8 | Map comparison between the conventional and ultrasonically excited maps for the pentameric C-reactive protein. To illustrate artifacts in the map obtained from the conventional sample of the pentameric C-reactive protein, top views (left) for the conventional (top, in grey) and ultrasonically excited (bottom, in green) samples are shown. Details of these maps reveal artifacts in the conventional map as a result of preferred orientation. Further, to demonstrate high-resolution and interpretability of the cryo-EM map shown in the bottom panel, a model (PDB ID: 7PKD[3]) is docked and jiggle-fitted within the map. Several sections of the amino acid backbone are shown (right). All maps are displayed with a level at 7.5σ above the mean.

4 | Data processing and analysis – hemagglutinin

High-resolution micrographs of a conventional cryo sample of hemagglutinin as well as a sample that was subjected to ultrasonic excitation were collected at the Dubochet Center for Imaging in Lausanne, using a Thermo Fisher Titan Krios G4i transmission electron microscope equipped with a Falcon IV direct electron detector. Single-particle reconstructions were performed using CryoSPARC v4.7.1[2], with the corresponding workflows illustrated in SI Figure 9 and 10. The data acquisition parameters and processing statistics are summarized in SI Table 3.

Micrographs were subjected to patch motion correction and patch CTF estimation. Micrographs with an estimated resolution of worse than 10 Å were rejected. Particles from the conventional and ultrasonically excited samples were picked using a blob picker with a diameter ranging from either 50 to 150 Å (conventional sample) or 80 to 150 Å (ultrasonically excited sample). The particles were extracted with a box size of 384 px and initially Fourier-cropped to 96 px.

For the conventional dataset, particles were first subjected to four rounds of 2D classification followed by *ab initio* reconstruction (three classes, C_1 symmetry) and then two rounds of heterogeneous refinement (all three *ab initio* classes, C_3 symmetry). Particles from the best class resulting from the final round of heterogeneous refinement were re-extracted with a box size of 384 px and non-uniformly refined (C_3 symmetry) producing the final volume shown in SI Figure 9.

Particles from the ultrasonically excited dataset were first subjected to three rounds of 2D classification. A comparison of the obtained 2D classes is shown in SI Figure 11 highlighting that ultrasonic excitation populates side views that are absent in the conventional dataset. Following 2D classification, top/bottom views and side views of the hemagglutinin trimer were separated. *Ab initio* reconstruction (C_1 symmetry) was first performed on a subset of particles containing predominantly top/bottom views producing a truncated hemagglutinin trimer. Separately, *ab initio* reconstruction (C_1 symmetry) was performed on particles representing predominantly side views producing a volume closely resembling the hemagglutinin trimer. Particles from this second *ab initio* reconstruction were then subjected to homogeneous refinement (C_3 symmetry), followed by two rounds of heterogeneous refinement (three classes, C_3 symmetry) using the previously homogeneously refined volume and two decoys. Particles

from the best class from the final round of heterogeneous refinement were then subjected to homogeneous refinement (C_3 symmetry) and subsequently re-extracted with a box size of 384 px. All originally selected particles were then heterogeneously refined (C_3 symmetry) against five copies of the *ab initio* reconstruction derived from the top/bottom views and one copy of a hemagglutinin volume derived from the side views. Particles from the best class were then homogeneously refined (C_3 symmetry) and subjected to a further round of heterogeneous refinement (C_3 symmetry) using three volumes: one copy of the *ab initio* reconstruction derived from the top/bottom views, and two copies of the complete hemagglutinin volume generated in the previous round of homogeneous refinement. Particles from the best class resulting from the final round of heterogeneous refinement were non-uniformly refined (C_3 symmetry) producing the final volume shown in Figure 3b and SI Figure 10. Here, best class refers to the reconstruction with no indicators of directional anisotropy (e.g. missing and density). Nevertheless, to enable a comparison of angular distributions after data processing, the other two classes in the final round of heterogeneous refinement were also re-extracted and non-uniformly refined and are displayed in SI Figure 10.

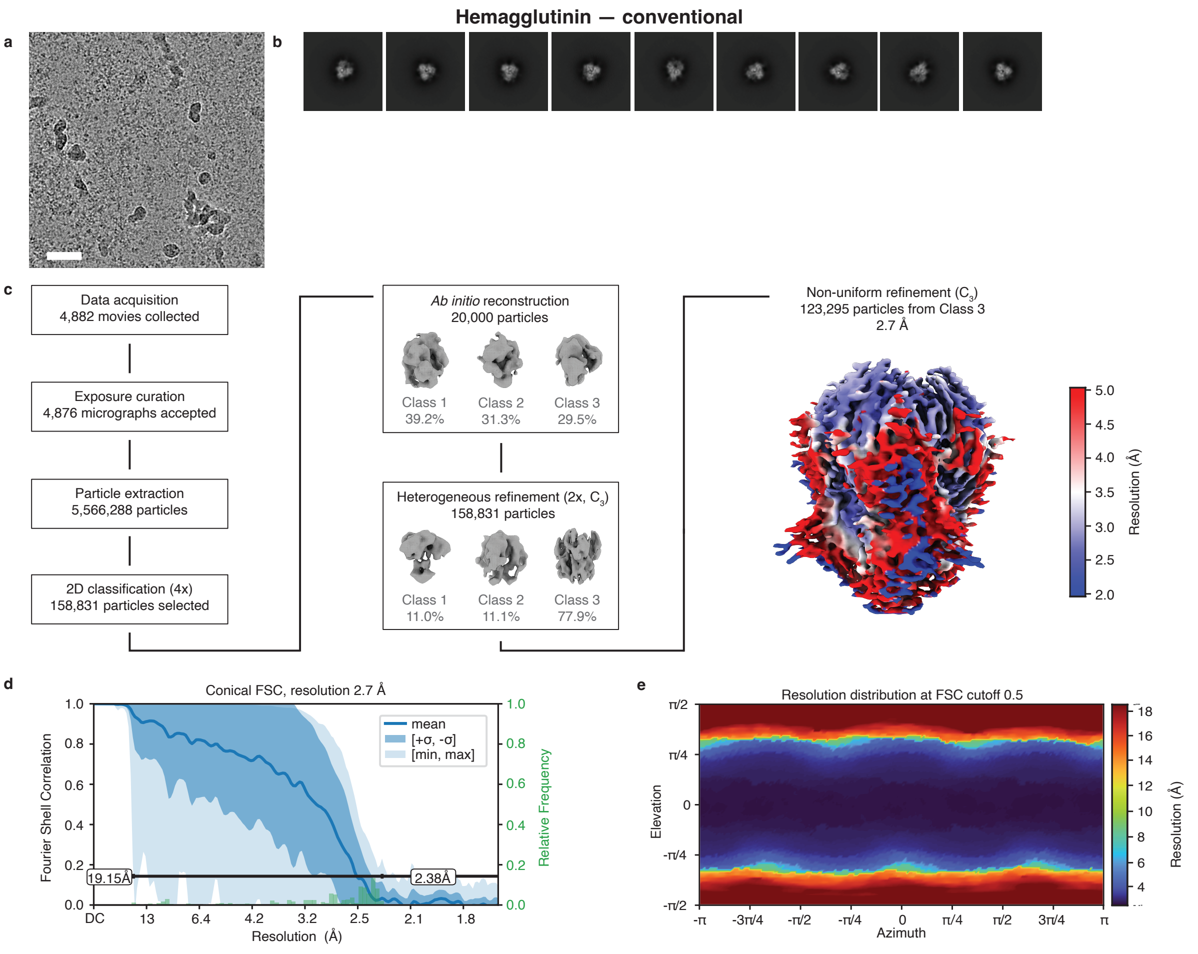

Figure S9 | Cryo-EM processing workflow for the conventional sample of hemagglutinin. **a** Representative micrograph. Scale bar, 500 Å. **b** Selected 2D class averages. **c** Data processing workflow in CryoSPARC. Symmetry imposed is indicated in parentheses. All volumes are displayed at a level of 10σ above the mean. The final map is colored according to the estimated local resolution at the FSC cutoff of 0.5. **d** Conical FSC, with the 0.143 threshold indicated by a black line. The mean FSC value is shown as a solid blue line. Dark blue shading indicates one standard deviation of the FSC curve, while the light blue shading represents the minimum and maximum FSC values. A histogram of the resolution values obtained from the conical FSC is shown in green. **e** Angular distribution of the resolution at FSC cutoff 0.5, generated using the 3DFSC file from Orientation Diagnostics job in CryoSPARC.

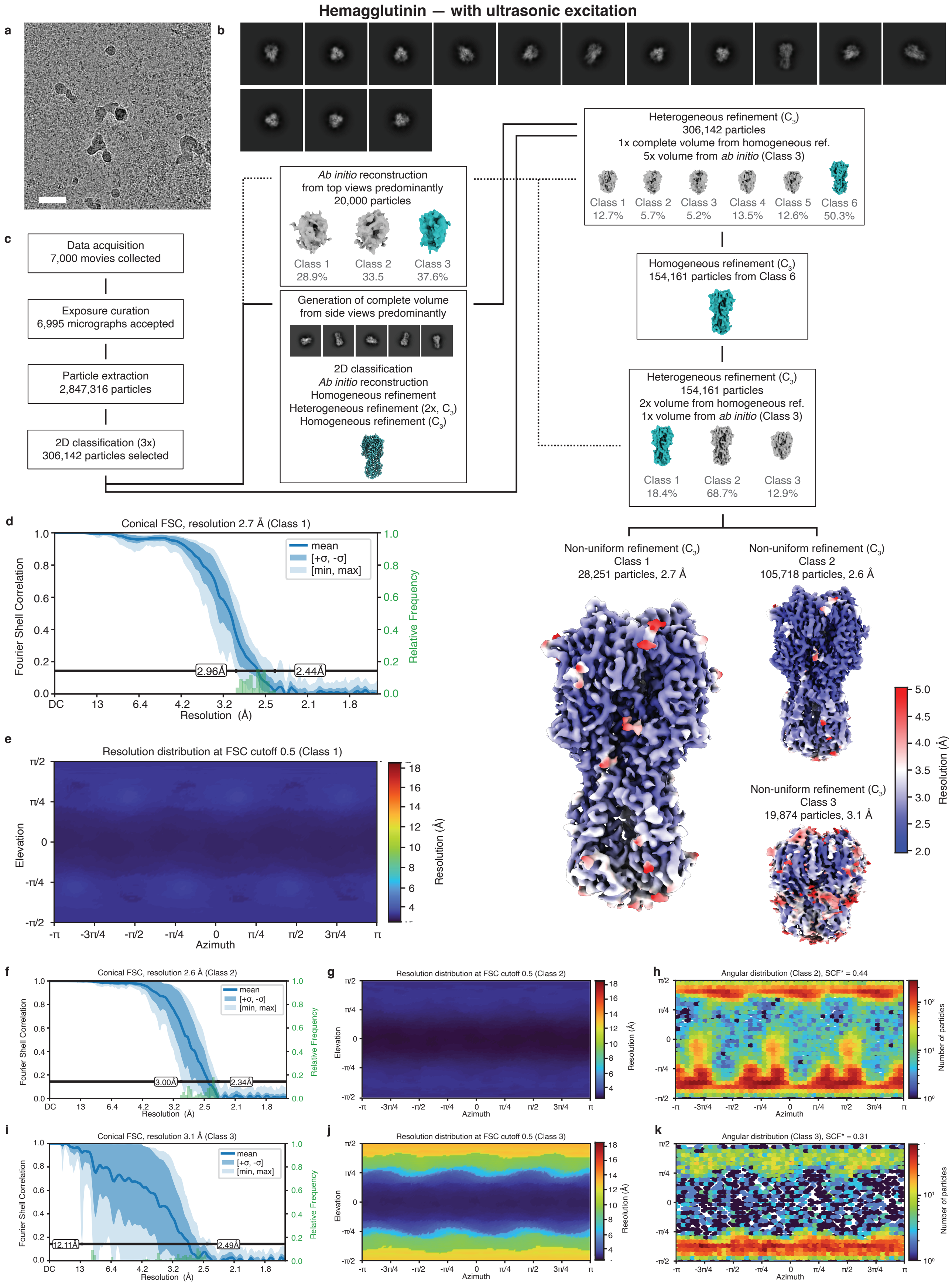
Hemagglutinin — with ultrasonic excitation
a
b
c
Data acquisition
7,000 movies collected
Exposure curation
6,995 micrographs accepted
Particle extraction
2,847,316 particles
2D classification (3x)
306,142 particles selected
Ab initio reconstruction
from top views predominantly
20,000 particles
Class 1
28.9%
Class 2
33.5
Class 3
37.6%
Generation of complete volume
from side views predominantly
2D classification
Ab initio reconstruction
Homogeneous refinement
Heterogeneous refinement (2x, C3)
Homogeneous refinement (C3)
Heterogeneous refinement (C3)
306,142 particles
1x complete volume from homogeneous ref.
5x volume from ab initio (Class 3)
Class 1 12.7%
Class 2 5.7%
Class 3 5.2%
Class 4 13.5%
Class 5 12.6%
Class 6 50.3%
Homogeneous refinement (C3)
154,161 particles from Class 6
Heterogeneous refinement (C3)
154,161 particles
2x volume from homogeneous ref.
1x volume from ab initio (Class 3)
Class 1 18.4%
Class 2 68.7%
Class 3 12.9%
Non-uniform refinement (C3)
Class 1
28,251 particles, 2.7 Å
Non-uniform refinement (C3)
Class 2
105,718 particles, 2.6 Å
Non-uniform refinement (C3)
Class 3
19,874 particles, 3.1 Å
Resolution (Å)
d
Conical FSC, resolution 2.7 Å (Class 1)
mean
[+σ, -σ]
[min, max]
2.96Å
2.44Å
Fourier Shell Correlation
Relative Frequency
Resolution (Å)
e
Resolution distribution at FSC cutoff 0.5 (Class 1)
Elevation
Azimuth
Resolution (Å)
f
Conical FSC, resolution 2.6 Å (Class 2)
3.00Å
2.34Å
g
Resolution distribution at FSC cutoff 0.5 (Class 2)
h
Angular distribution (Class 2), SCF* = 0.44
Number of particles
i
Conical FSC, resolution 3.1 Å (Class 3)
12.11Å
2.49Å
j
Resolution distribution at FSC cutoff 0.5 (Class 3)
k
Angular distribution (Class 3), SCF* = 0.31

Figure S10 | Processing workflow for the ultrasonically excited sample of hemagglutinin. **a** Representative micrograph. Scale bar, 500 Å. **b** Selected 2D class averages. **c** Data processing workflow in CryoSPARC. Symmetry imposed is indicated in parentheses. All volumes are displayed at a level of 10σ above the mean. Final maps are colored according to the estimated local resolution at the FSC cutoff of 0.5. **d, f, i** Conical FSC, with the 0.143 threshold indicated by a black line. The mean FSC value is shown as a solid blue line. Dark blue shading indicates one standard deviation of the FSC curve, while the light blue shading represents the minimum and maximum FSC values. A histogram of the resolution values obtained from the conical FSC is shown in green. **e, g, j** Angular distribution of the resolution at FSC cutoff 0.5, generated using the 3DFSC file from Orientation Diagnostics job in CryoSPARC. **h, k** Orientation distribution of the particles in the indicated final volume.

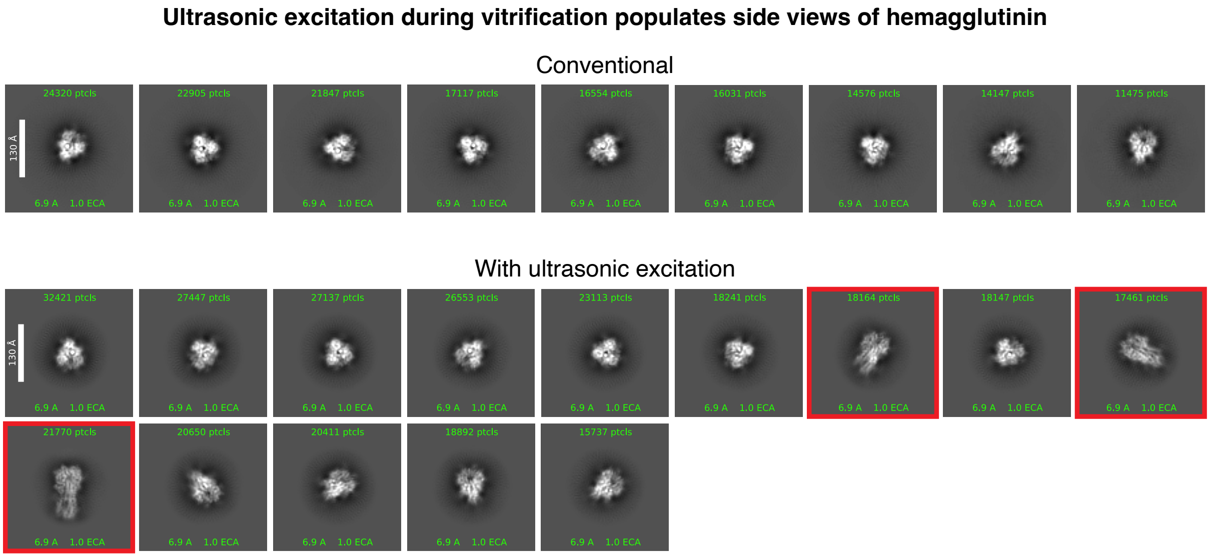

Figure S11 | Ultrasonic excitation during vitrification populates side views of hemagglutinin. With ultrasonic excitation, the 2D class averages show side views (bottom, highlighted in red), which are missing in a conventional sample (top).

5 | Estimate of the angular distribution in the conventional and ultrasonically excited hemagglutinin samples

Figure 3 compares reconstructions of hemagglutinin from a conventional sample as well as a sample that was subjected to ultrasonic excitation during vitrification, with the corresponding workflows for obtaining the reconstructions detailed in Supplementary Information 4. The angular distributions displayed in Fig. 3 represent the distributions of the particles that contribute to the maps displayed. However, because hemagglutinin has such strong preferred orientation, these distributions likely do not accurately reflect the angular distribution of all particles in the sample. The conventional dataset only yielded a truncated reconstruction, so that the particle orientations determined against this map likely have large errors. For the sample vitrified with ultrasonic excitation, the workflow that yields volume shown in the main text removes a large number of top and bottom views during the heterogeneous refinement steps, so their population is likely underrepresented in the angular distribution.

To arrive at a more realistic estimate of the angular distribution, we therefore refined all particles picked after 2D classification against the complete volume of hemagglutinin shown in Fig. 3b. The corresponding workflows are illustrated in Fig. S12a. For the conventional sample, this produces a volume that more closely resembles the hemagglutinin trimer but still exhibits a large amount of directional anisotropy. The corresponding angular distribution is largely dominated by top and bottom views and has an SCF* value of 0.43 (Fig. S12b). For the sample vitrified with ultrasonic excitation, the same process yields an angular distribution that additionally features a significant population of side views and has an SCF* of 0.56. Note, this procedure for estimating the angular distribution of the sample likely includes a considerable fraction of damaged particles in the statistics that would otherwise be removed through heterogeneous refinement. Such particles likely have a large error in the angular assignment, which has the effect of artificially increasing the SCF* value.

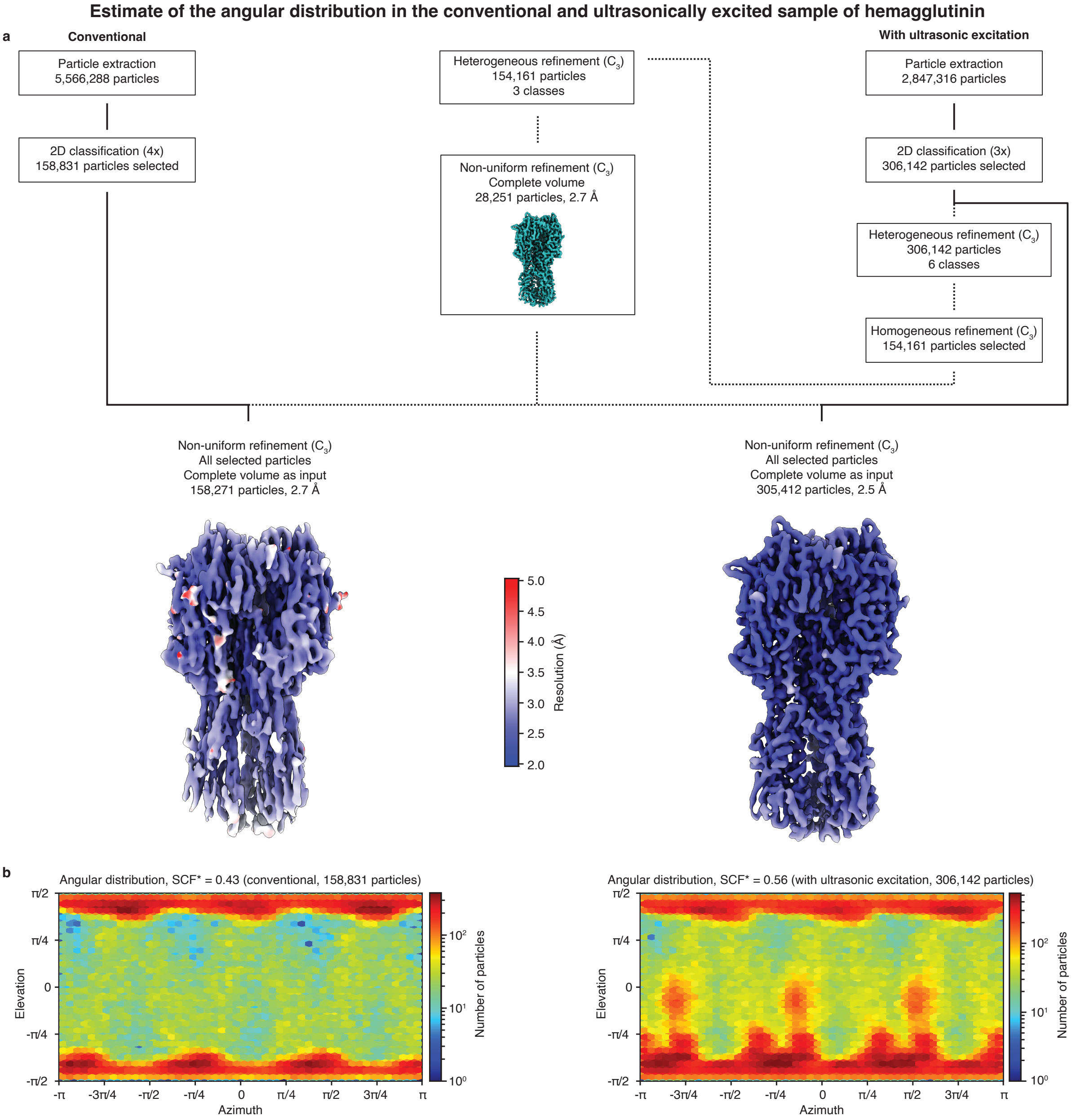

Figure S12 | Estimate of the angular distribution in the conventional and ultrasonically excited sample of hemagglutinin. a Data processing workflow in CryoSPARC. All maps are displayed at the level 10σ above the mean. Symmetry imposed is indicated in parentheses. The final map is displayed with the local resolution estimation indicated in color. **b** Estimate of the angular distribution obtained from a non-uniform refinement of all selected particles against the complete hemagglutinin volume from Fig. 3b.

6 | Cryo-EM data collection and processing statistics

Supplementary Table 1: Data collection parameters and processing statistics for the 50S ribosomal subunit.

	50S conventional sample (EMD-54830)	50S ultrasonically excited sample (EMD-54829)
**Data collection and processing**		
Microscope	Titan Krios G4i	Titan Krios G4i
Camera	Falcon 4i	Falcon 4i
Energy filter	N/A	N/A
Magnification	96,000	96,000
Voltage (kV)	300	300
Electron exposure (e^{-}/Å^2)	50	50
Defocus range (µm)	-0.4 – -1.2	-0.4 – -1.2
Pixel size (Å)	0.830	0.830
Image format	EER	EER
Symmetry imposed	C_1	C_1
Initial particle images (no.)	238,747	625,311
Final particle images (no.)	50’000	50’000
Map resolution (Å)	2.8	2.8
FSC threshold	0.143	0.143
Map resolution range (Å)	3.18 – 2.46	3.06 – 2.61
Map sharpening *B* factor (Å^2)	29.7	33.0
Orientation diagnostics		
cFAR	0.35	0.58
SCF*	0.66	0.83

Supplementary Table 2: Data collection parameters and processing statistics for C-reactive protein.

	C-reactive protein pentamer conventional sample (EMD-54834)	C-reactive protein pentamer ultrasonically excited sample (EMD-54833)	C-reactive protein decamer conventional sample (EMD-54836)	C-reactive protein decamer ultrasonically excited sample (EMD-54835)
**Data collection and processing**				
Microscope	Titan Krios G4i	Titan Krios G4i	Titan Krios G4i	Titan Krios G4i
Camera	Falcon 4i	Falcon 4i	Falcon 4i	Falcon 4i
Energy filter	N/A	N/A	N/A	N/A
Magnification	96,000	96,000	96,000	96,000
Voltage (kV)	300	300	300	300
Electron exposure ($e^{-}/Å^2$)	50	50	50	50
Defocus range (μm)	-1.0 – -2.4	-1.0 – -2.4	-1.0 – -2.4	-1.0 – -2.4
Pixel size (Å)	0.830	0.830	0.830	0.830
Image format	EER	EER	EER	EER
Symmetry imposed	C_5	C_5	C_1	C_1
Initial particle images (no.)	5,448,069	3,905,203	5,448,069	3,905,203
Final particle images (no.)	30'000	30'000	50'000	50'000
Map resolution (Å)	4.3	3.7	3.3	3.2
FSC threshold	0.143	0.143	0.143	0.143
Map resolution range (Å)	4.63 – 3.61	3.93 – 3.42	3.51 – 3.01	3.35 – 2.88
Map sharpening *B* factor ($Å^2$)	147.6	118.3	78.2	66.1
Orientation diagnostics				
cFAR	0.47	0.64	0.68	0.67
SCF*	0.80	0.90	0.89	0.90

Supplementary Table 3: Data collection parameters and processing statistics for hemagglutinin.

	Hemagglutinin conventional sample (EMD-54832)	Hemagglutinin ultrasonically excited sample (EMD-54831)
**Data collection and processing**		
Microscope	Titan Krios G4i	Titan Krios G4i
Camera	Falcon 4i	Falcon 4i
Energy filter	N/A	N/A
Magnification	96,000	96,000
Voltage (kV)	300	300
Electron exposure ($e^-/Å^2$)	50	50
Defocus range (μm)	-1.0 – -2.4	-1.0 – -2.4
Pixel size (Å)	0.830	0.830
Image format	EER	EER
Symmetry imposed	C_3	C_3
Initial particle images (no.)	5,566,288	2,847,316
Final particle images (no.)	123’295	28’251
Map resolution (Å)	2.7	2.7
FSC threshold	0.143	0.143
Map resolution range (Å)	19.15 – 2.38	2.96 – 2.44
Map sharpening *B* factor ($Å^2$)	77.0	66.1
Orientation diagnostics		
cFAR	0.00	0.58
SCF*	0.25	0.81

7 | References

1. Haubner, M., Hruby, J., Straub, M.S., Williams, H.M., Drabbels, M. & Lorenz, U.J. *Manuscript in preparation.* (2025)
2. Punjani, A., Rubinstein, J., Fleet, D.J. & Brubaker, M.A. cryoSPARC: algorithms for rapid unsupervised cryo-EM structure determination. Nat. Methods. **14**, 290-296 (2017).
3. Noone, D.P., van der Velden, T.T. & Sharp, T.H. Cryo-Electron Microscopy and Biochemical Analysis Offer Insights Into the Effects of Acidic pH, Such as Occur During Acidosis, on the Complement Binding Properties of C-Reactive Protein. Front. Immunol. **12**, 757633 (2021).
